# Laryngoscopy

**Published:** 1867-11

**Authors:** H. A. Johnson

**Affiliations:** 611 Wabash Avenue, Chicago


### LARYNGOSCOPY.

Mr. Editor:—I have frequently been able to overcome the irritability of the throat, sometimes so troublesome in laryngoscopic examinations, by throwing upon the velum and posterior portions of the pharynx a spray of sulphuric ether, by means of Richardson's apparatus. The patient should take a full inspiration before commencing the operation, and the spray should be rapidly carried from point to point, so as not to produce congelation. This method is quicker, more convenient, and more efficacious than ice.

611 Wabash Avenue, *Chicago, Oct. 1, 1867.* H. A. JOHNSON.

